# Fatigue Life Prediction of FRP-Strengthened Reinforced Concrete Beams Based on Soft Computing Techniques

**DOI:** 10.3390/ma18020230

**Published:** 2025-01-07

**Authors:** Zhimei Zhang, Xiaobo Wang

**Affiliations:** Department of Civil Engineering, School of Mechanics and Engineering Science, Shanghai University, Shanghai 200444, China; zhangzhimei@staff.shu.edu.cn

**Keywords:** multi-objective genetic algorithm evolutionary polynomial regression, gene expression programming, fatigue life, prediction model, fiber-reinforced polymer composite material, FRP strengthened beams

## Abstract

This paper establishes fatigue life prediction models using the soft computing method to address insufficient parameter consideration and limited computational accuracy in predicting the fatigue life of fiber-reinforced polymer (FRP) strengthened concrete beams. Five different input forms were proposed by collecting 117 sets of fatigue test data of FRP-strengthened concrete beams from the existing literature and integrating the outcomes from Pearson correlation analysis and significance testing. Using Gene Expression Programming (GEP), the effects of various input configurations on the accuracy of model predictions were examined. The model prediction results were also evaluated using five statistical indicators. The GEP model used concrete compressive strength, the steel reinforcement stress range ratio to the yield strength, and the stiffness factor as input parameters. Subsequently, using the same input parameters, the Multi-Objective Genetic Algorithm Evolutionary Polynomial Regression (MOGA-EPR) method was then employed to develop a fatigue life prediction model. Sensitivity analyses of the GEP and MOGA-EPR models revealed that both could precisely capture the fundamental connections between fatigue life and multiple contributing variables. Compared to existing models, the proposed ones have higher prediction accuracy with a coefficient of determination reaching 0.8, significantly enhancing the accuracy of fatigue life predictions for FRP-strengthened concrete beams.

## 1. Introduction

Fiber-reinforced polymers (FRP), which possess benefits such as lightweight, high strength, corrosion and fatigue resistance, and ease of construction [1,2], have been increasingly used in the reinforcement of bridge structures over the past few years [3]. In addition, FRP has also been used for the reinforcement of masonry structures. Gattulli et al. [4] evaluated the effectiveness of FRP reinforcement in masonry buildings by determining the vulnerability curves. The FRP bending reinforcement of reinforced concrete beams mainly consists of two forms: external bonding (EB) and near-surface mounted (NSM) reinforcement. External bonding involves attaching the FRP material to the bottom of the reinforced concrete beam, while near-surface mounted reinforcement involves embedding the FRP material into a groove or channel near the surface of the beam, allowing it to integrate closely with the beam’s main structure. Whether external bonding or near-surface mounted reinforcement, the use of FRP materials can effectively delay crack development, improve the stiffness of the component, and thus extend the fatigue life of the bending member.

Fatigue life, an essential indicator of structural reliability, is the basis for the fatigue-resistant design of bridge structures. However, the fatigue characteristics of FRP-strengthened concrete girders are influenced by numerous factors, such as FRP–concrete interfacial bond performance, the range of stress changes in reinforcement, crack extension, etc., which makes the prediction of fatigue life very complicated. Many researchers have carried out comprehensive experimental studies and theoretical analyses, which have led to the development of multiple fatigue life prediction models for use in civil engineering applications. Aidoo et al. [5] and Barnes et al. [6] discovered that the lifespan of beams strengthened using FRP is primarily influenced by the performance of longitudinal tensile steel reinforcement under fatigue, as evidenced by fatigue testing. Shahawy et al. [7] discovered that adding more layers of CFRP fabric reinforcement significantly enhances the longevity of reinforced beams under fatigue conditions, as demonstrated by fatigue tests. Zhang et al. [8] determined through testing that concrete compressive strength significantly influences the fatigue behavior of reinforced components and that the fatigue life of reinforced beams is greatly extended by employing higher-strength concrete. In conclusion, the primary failure mode of FRP-strengthened reinforced concrete beams is the fracture of longitudinal tensile steel bars due to the continuous accumulation of fatigue damage. The key parameters influencing the fatigue performance are the stress amplitude of the steel bars, the concrete strength, and the amount of FRP reinforcement. Charalambidi et al. [9] summarized the other literature, indicating that when the steel reinforcement stress range is the same, the ratio of maximum steel stress to yield strength is consistent, and the ratio of longitudinal tensile steel reinforcement axial stiffness to FRP axial stiffness significantly influences the fatigue behavior of FRP-strengthened beams. Papakonstantinou et al. [10] developed a fatigue life prediction model using fatigue test data from 24 GFRP-reinforced and unreinforced beams, where the steel reinforcement stress range was used as a variable. Dong et al. [11] observed that the fatigue damage forms of the reinforced beams changed when they applied various fatigue loads to ten identical CFRP-strengthened beams. Steel fracture and CFRP debonding damage were found to be the two primary forms of fatigue damage in reinforced beams; as the upper limit of the fatigue load increased, the damage form shifted from rebar fatigue fracture to CFRP debonding damage. The fatigue life prediction model was created by combining this data with experimental data from the other literature where the steel reinforcement stress range was used as a variable. Sun et al. [12] statistically analyzed the collected test data. They found more pronounced differences in the statistical characteristics of the data with the stress range $\sigma_{r}=175\;\mathrm{MPa}$ as the boundary and accordingly established a segmented functional fatigue life prediction model.

The models developed by various scholars based on their respective experimental studies primarily reflect the impact of steel reinforcement stress range on fatigue life. Nevertheless, because of the intricate nature of fatigue testing and the inconsistency of the outcomes, these models not only vary in parameter values and applicable ranges but also neglect the influence of other factors. Therefore, it is essential to establish a more accurate and universally applicable fatigue life prediction model for FRP-strengthened beams that comprehensively considers the influence of all significant factors.

In recent years, many scholars have attempted to utilize various machine learning algorithms to help predict the performance of FRP-strengthened components using extensive experimental data, achieving good predictive results [13,14,15,16]. However, most machine learning algorithms generate black-box models that cannot provide explicit mathematical expressions. In contrast, Gene Expression Programming (GEP) possesses a robust function discovery capability, providing explicit expressions of prediction models and offering model interpretability and ease of application. Evolutionary Polynomial Regression with Multi-Objective Genetic Algorithms (EPR-MOGA) generates regression models that are both accurate and concise by simultaneously optimizing prediction accuracy and model complexity, effectively preventing overfitting.

In light of this, the study gathered 117 data sets from bending fatigue tests conducted on FRP-strengthened concrete beams. The data was then subjected to Pearson correlation analysis and significance testing to determine the parameters that significantly relate to the fatigue life. According to these findings, GEP was employed to examine the experimental data concerning fatigue life and its primary determining variables, establishing a fatigue life prediction model for FRP-strengthened concrete beams that take into account factors such as steel reinforcement stress range, concrete compressive strength, and the amount of FRP reinforcement. Subsequently, MOGA-EPR was employed with the same input parameters to develop a fatigue life prediction model. Finally, a comparative analysis with existing fatigue life prediction models is conducted to verify the precision and dependability of the proposed prediction model in this paper.

## 2. Algorithm Introduction

### 2.1. GEP

Gene Expression Programming (GEP), proposed by Ferreira [17], is a program for evolution developed on the basis of genetic programming (GP). Compared with GP, GEP has higher coding and manipulation flexibility, more vital function discovery ability [18], and can effectively model complex nonlinear problems.

The building blocks of chromosomes are called genes, and they are primarily composed of two parts: the head, which can have function symbols as well as terminal symbols, and the tail, which only has terminal symbols. The function expression corresponding to a gene can model the complex relationships between variables by combining function symbols and terminal symbols. The length of the gene head *h* can be predetermined based on the specific problem, while the size of the tail *t* is calculated using the following formula:

(1) $$t=h\times \left(n-1\right)+1.$$

In the formula, *n* represents the maximum number of operations in the function set.

**Figure 1:**
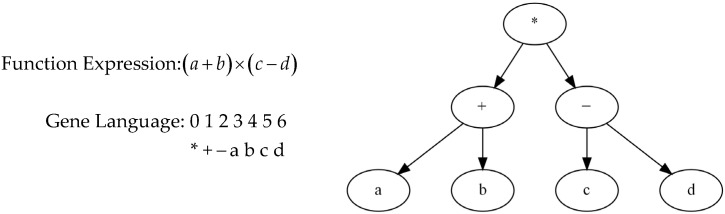
Chromosome encoding method.

There are two different kinds of languages in GEP: expression tree language and gene language. The expression tree language is derived by decoding the gene language. Gene decoding involves reading the linear string of genotype encoding from left to right and arranging the characters according to the corresponding hierarchical order and syntactic rules to form the tree structure of the expression. Figure 1 illustrates the chromosome encoding method in gene expression programming, where the linear encoding string is decoded into an expression tree. The root of the tree is the multiplication operator, the left subtree has the addition operator as its root with a and b as its child nodes, and the right subtree has the subtraction operator as its root with c and d as its child nodes. The final expression is $\left(a+b\right)\times \left(c-d\right)$, fully demonstrating the process of how gene language is transformed into expression tree language.

Fitness functions play a crucial role in the evolution of GEP. Generally, the higher the fitness, the closer the chromosome is to the optimal solution. The GEP algorithm has three fitness calculation functions: the absolute error fitness function, the relative error-based fitness function, and the fitness function used for logic synthesis problems. Among these, the Root Mean Square Error (*RMSE*) is a fitness function with high robustness, suitable for optimization problems involving continuous variables. Therefore, the *RMSE* function is used as the fitness function in this paper. The formula for calculating the fitness in this paper is shown in Equation (2).

(2) $$f_{i}=\frac{1000}{1+RMSE_{i}},$$

where $f_{i}$ denotes the fitness of chromosome *i*, and $RMSE_{i}$ denotes the root mean square error for chromosome *i*.

### 2.2. MOGA-EPR

Giustolisi and Savic [19] proposed the multi-objective genetic algorithm evolutionary polynomial regression method (MOGA-EPR). This method combines genetic algorithms with the least squares regression approach. Using a multi-objective genetic algorithm, it starts by conducting a global search for the optimal expressions between input and output data. Once the expression structure is identified through evolution, the model parameters are estimated by employing the least squares method in order to arrive at the final expression.

Equation (3) is the general form of the expression [20]:

(3) $$Y=a_{0}+\sum_{j=1}^{m}a_{j}\times {\left(X_{1}\right)}^{ES(j,1)}\ldots {\left(X_{k}\right)}^{ES(j,k)}\times f\left({\left(X_{1}\right)}^{ES(j,k+1)}\ldots {\left(X_{k}\right)}^{ES(j,2k)}\right).$$

In the formula, $a_{j}$ representing the parameter values; *k* is the number of input variables; $X_{i}$ is the input vectors; f is a custom function; $\mathrm{ES}\left(j,i\right)$ is the exponent of the *i*th input within the *j*th term of the polynomial; m is the number of terms in the polynomial, excluding the bias $a_{0}$.

In this expression, the exponent of the input variables for each term is determined by the matrix ${\mathrm{ES}}_{m\times k}$. First, a set of possible exponent values (including 0) is selected. MOGA-EPR searches the possible exponent matrices to find the optimal input combination, constructing a suitable polynomial model. If, during the search, some of the assigned exponents are 0, the corresponding input variables will be eliminated from that term, thus simplifying the model. For more information on EPR-MOGA, see references [19,20,21,22].

## 3. Model Development

### 3.1. Methodology

**Figure 2:**
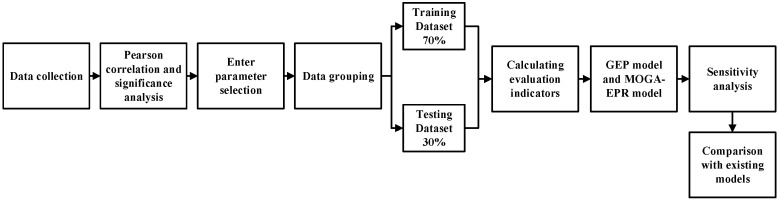
Methodology flowchart of the current study.

Figure 2 displays the flowchart illustrating the techniques and processes utilized in this paper. The process begins with the collection of data, which is then followed by Pearson correlation and significance analysis. Next, input parameters are selected, and the data is grouped accordingly. Statistical metrics are then calculated, and a model is developed. A sensitivity analysis is conducted, and finally, the results of the model proposed in this paper are compared with those of existing models.

### 3.2. Data Analysis

**Figure 3:**
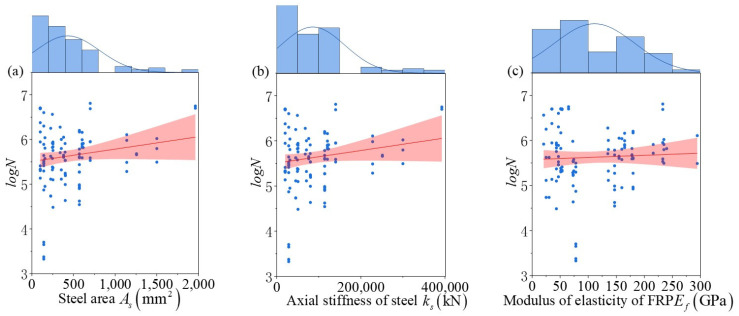

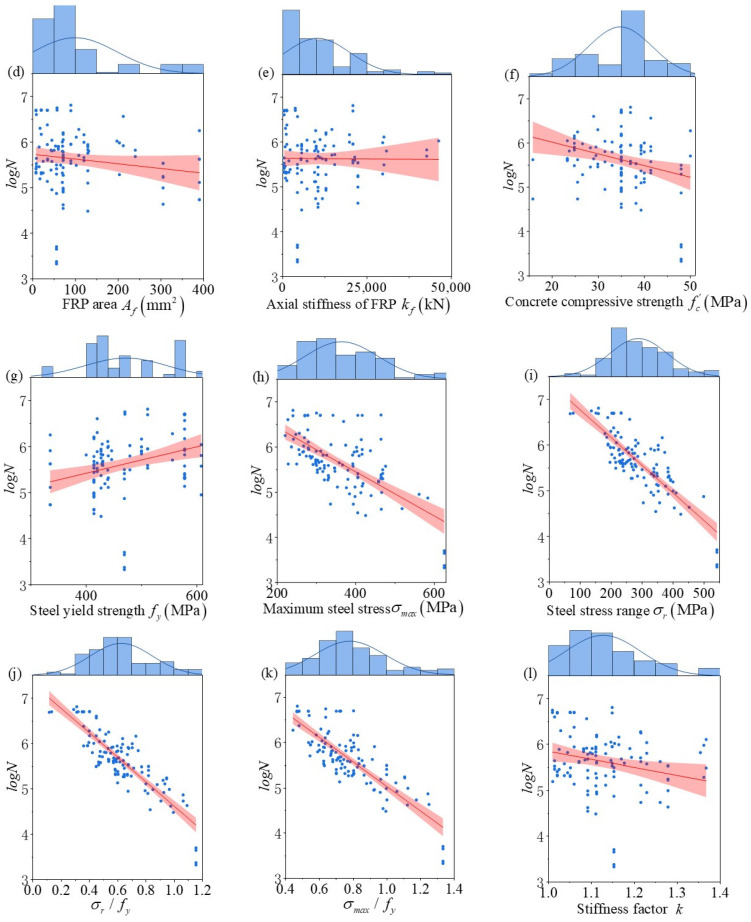
Histogram marginal plot of the link between fatigue life and various parameters.

Data were collected from 21 papers [5,6,10,11,23,24,25,26,27,28,29,30,31,32,33,34,35,36,37,38,39]. Due to variations in the reinforcement materials and failure modes used in different experiments, the main criteria for data selection were all were FRP-strengthened beams, limited to reinforcements including CFRP, GFRP, and AFRP; the reinforced beams did not undergo corrosion; the failure mode was rebar fatigue fracture. The final number of samples was 117 sets, of which 94 were externally bonded FRP-strengthened beams, and 23 were near-surface-mounted FRP-strengthened beams. The test data on the main factors influencing fatigue life were statistically analyzed, and the results are shown in Table 1. The relationship between the parameters in the dataset and the fatigue life is illustrated in Figure 3. A combination of both reveals that the dataset is highly dispersed, encompasses a broad range of values, and has a relatively even distribution of parameters, thus providing a reliable data foundation for establishing a fatigue life prediction model.

In the table, *E_s_*, *A_s_*, and *k_s_* = *E_s_A_s_* represent the elastic modulus of longitudinal tensile steel reinforcement, cross-sectional area, and axial stiffness; because the range of changes in Es is relatively small, this paper in the analysis of the unified 200 GPa; *A_f_*, *E_f_*, and *k_f_* = *E_f_A_f_* represent the cross-sectional area, modulus of elasticity, and axial stiffness of the FRP; *f’_c_* represents the compressive strength of concrete, *f_y_* represents the yield strength of steel reinforcement; *σ_max_*, *σ_min_*, and *σ_r_* = *σ_max_* − *σ_min_* represent the longitudinal tensile steel reinforcement maximum stress, minimum stress, stress range; *σ_max_*/*f_y_*, and *σ_r_*/*f_y_* represent the ratio of maximum stress to yield strength and the ratio of stress range to yield strength in longitudinal tensile steel reinforcement, respectively; *k* = (*k_s_* + *k_f_*)/*k_s_* represents the stiffness factor, and *logN* represents the logarithmic fatigue life.

**Figure 4:**
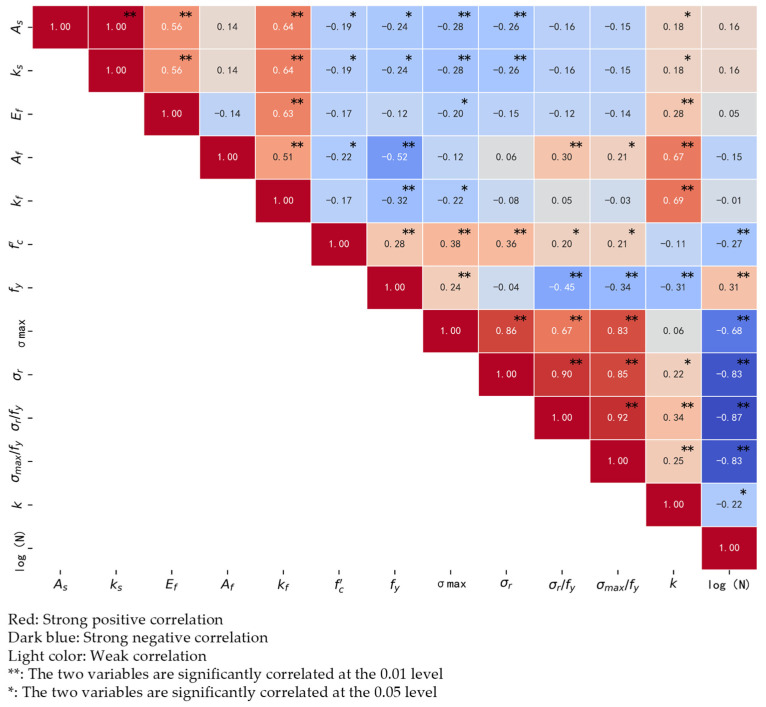
Pearson correlation coefficient heatmap.

To further clarify the relationship between fatigue life and the parameters in Table 1, Pearson correlation analysis and significance testing were conducted on the database. The Pearson correlation coefficient is widely used in data exploration to help identify potential linear relationships in the data. The value of the Pearson correlation coefficient ranges from −1 to 1. A value closer to 1 indicates a stronger positive correlation, a value closer to −1 indicates a stronger negative correlation, and a value close to 0 indicates no linear correlation between the two variables. Significance testing is used to determine if these coefficients are statistically significant; the Pearson correlation coefficient is statistically significant only when the significance level is less than 0.05, indicating that the observed association is not the result of chance. Figure 4 displays the findings of the analysis, where ** denotes a significant linear correlation between the two variables at the 0.01 level and * denotes a significant linear correlation between the two variables at the 0.05 level. As seen in Figure 4, the fatigue life of FRP-strengthened beams shows a significant correlation at the 0.01 level with concrete compressive strength, steel yield strength, and steel reinforcement stress range, and important at the 0.05 level with stiffness factor. Therefore, these significantly correlated values may be entered into the model as input parameters. The parameters with substantial correlations at the 0.01 level are selected, i.e., $logN=f\left(f_{c}^{\prime },f_{y},\sigma_{r}\right)$; the parameters with significant correlations at the 0.05 level are selected, i.e., $logN=f\left(f_{c}^{\prime },f_{y},\sigma_{r},k\right)$; the correlation coefficient between the ratio of steel reinforcement stress range to yield strength and the fatigue life is as high as 0.87, so it can be input as a parameter, i.e., $logN=f\left({f\prime }_{c},\sigma_{r}/f_{y},k\right)$.

### 3.3. Software Parameter Settings

**Figure 5:**
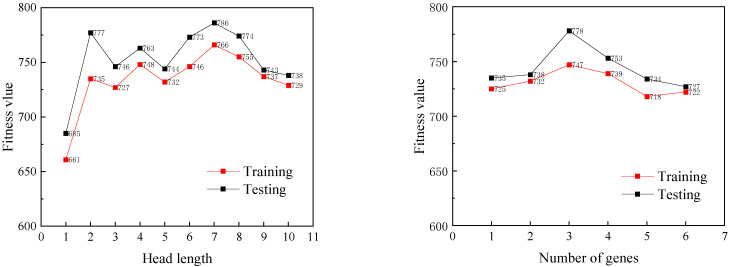
Determination of optimal parameters for the GEP algorithm.

A fatigue life prediction model for FRP-strengthened beams was developed using the program GeneXproTools 5.0. A 7:3 ratio was used to randomly separate the 117 data sets into training and test sets, yielding 82 training sets and 35 testing sets. The optimal values for parameters such as gene head length and gene number were established using a trial-and-error strategy, while the length of the gene tail was determined according to Equation (1). Figure 5 shows how the fitness value varies when head length and gene count vary. The values correlating to the most significant fitness value were determined to be the ideal values for both parameters. The ideal values for head length and gene number are 7 and 3, respectively. The function set F consists of all the function symbols that may be involved in the function expressions. After testing, the function set selected in this paper is $F=\left\{+,-,\times ,÷,\sqrt{x},\sqrt[{3}]{x},1/x,x^{2},x^{3}\right\}$. The values for other parameters are based on references [13], and particular parameters are enumerated in Table 2.

### 3.4. Selection of Optimal Input Form

This paper considers five distinct models when choosing model input parameters to determine the best input form and investigate the impact of changing input forms on the model’s prediction accuracy. Model 1 considers only the steel reinforcement stress range as a parameter. Model 2 is based on the research findings of Charalambides et al. [8]. The database’s Pearson correlation analysis findings determine the input parameters for Models 3, 4, and 5.

The five input forms were input into GeneXproTools 5.0 software, which was then run to obtain the corresponding prediction models. This paper uses five metrics to evaluate the feasibility and accuracy of each prediction scheme: the coefficient of determination ($R^{2}$), root mean square error (*RMSE*), mean absolute error (*MAE*), relative root mean square error (*RRSE*), and mean absolute percentage error (*MAPE*). The following are the formulae for these five assessment measures.

(4) $$R^{2}=\frac{{\left[\sum_{i=1}^{n}\left(logN_{ei}-\bar{logN_{e}}\right)\left(logN_{pi}-\bar{logN_{p}}\right)\right]}^{2}}{\sum_{i=1}^{n}{\left(logN_{ei}-\bar{logN_{e}}\right)}^{2}\sum_{i=1}^{n}{\left(logN_{pi}-\bar{logN_{p}}\right)}^{2}},$$

(5) $$RMSE=\sqrt{\frac{1}{n}\sum_{i=1}^{n}{\left(logN_{ei}-logN_{pi}\right)}^{2}},$$

(6) $$MAE=\frac{1}{n}\sum_{i=1}^{n}\left|logN_{ei}-logN_{pi}\right|,$$

(7) $$RRSE=\sqrt{\frac{\sum_{i=1}^{n}{\left(logN_{ei}-logN_{pi}\right)}^{2}}{\sum_{i=1}^{n}{\left(logN_{ei}-\bar{logN_{e}}\right)}^{2}}},$$

(8) $$MAPE=\frac{1}{n}\sum_{i=1}^{n}\left|\frac{logN_{ei}-logN_{pi}}{logN_{ei}}\right|,$$

where $logN_{ei}$ and $logN_{pi}$ are the experimental and predicted values of the logarithmic fatigue life, respectively, and $\bar{logN_{e}}$ is the average of the experimental values of the logarithmic fatigue life. $\bar{logN_{p}}$ is the average of the predicted values of the logarithmic fatigue life.

The results of the five input forms and the evaluation metrics are shown in Table 3. From Table 3, it is evident that Model 5 has a coefficient of determination as high as 0.8, which is higher than the other four models, and it also has the lowest values for root mean square error, mean absolute error, and other metrics. Thus, the combination of concrete compressive strength, the ratio of steel reinforcement stress range to yield strength, and the stiffness factor is the best input form for the model. Model 5 is named the GEP model.

**Figure 6:**
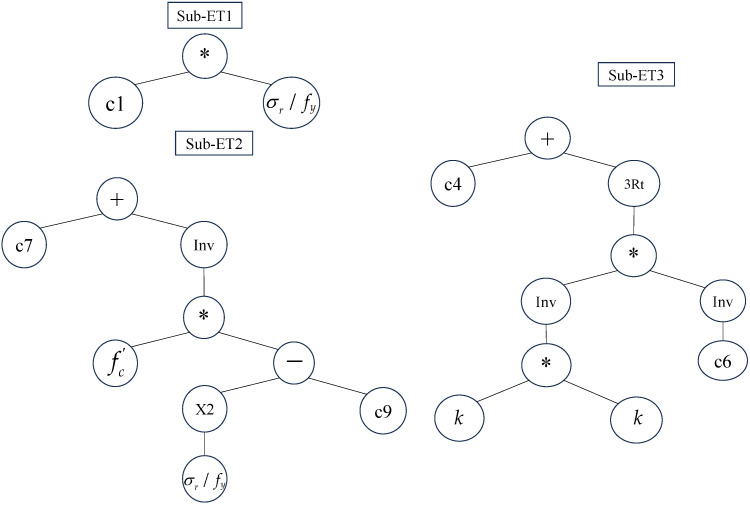
Expression tree.

Figure 6 shows the expression tree of the GEP model generated by the GeneXproTools 5.0 software. In Figure 6, Inv, X2, *, and 3Rt represent 1/x, $x^{2}$, ×, and $\sqrt[{3}]{x}$, respectively. The first gene contains one constant, $c_{1}$, which is −2.55; the second contains two constants, $c_{7}$, which is −0.626, and $c_{9}$, which is 1.37; the third contains two constants, $c_{4}$, which is 8.65 and $c_{6}$, which is −2. Since the connection function for the genes on the chromosome is “+”, the predictive function for the fatigue life of FRP-strengthened concrete beams can be derived as follows:

(9) $$logN=8.024-2.55\frac{\sigma_{r}}{f_{y}}+\frac{1}{f_{c}^{\prime }\left({\left(\frac{\sigma_{r}}{f_{y}}\right)}^{2}-1.37\right)}+\sqrt[{3}]{\frac{1}{-2k^{2}}}.$$

### 3.5. MOGA-EPR Model

Using the EPR-MOGA-XL tool to construct the MOGA-EPR model, we divide the dataset into training and test sets in a 7:3 ratio, with the input variable $f_{c}^{\prime },\sigma_{r}/f_{y},k$ and the output variable *logN*. In Equation (3), the number of input variables is 3, so in the EPR structure, *k* = 3. After testing, the maximum number of polynomial terms in the EPR structure, m, is set to 5. The candidate variables for the exponent matrix **ES** are [−2, −1, −0.5, 0, 0.5, 1, 2, 3]. The final MOGA-EPR model obtained is

(10) $$\begin{array}{ll} logN & =10.03-10.94\frac{\sigma_{r}}{f_{y}}\sqrt{k}-0.67\sqrt{f_{c}^{\prime }}{\left(\frac{\sigma_{r}}{f_{y}}\right)}^{3}k^{2}+0.78\sqrt{f_{c}^{\prime }}{\left(\frac{\sigma_{r}}{f_{y}}\right)}^{2}k\\ & -0.04\frac{\sqrt{f_{c}^{\prime }k}}{\sigma_{r}/f_{y}}+5.01{\left(k\frac{\sigma_{r}}{f_{y}}\right)}^{2} \end{array}.$$

### 3.6. Sensitivity Analysis of the Models

**Figure 7:**
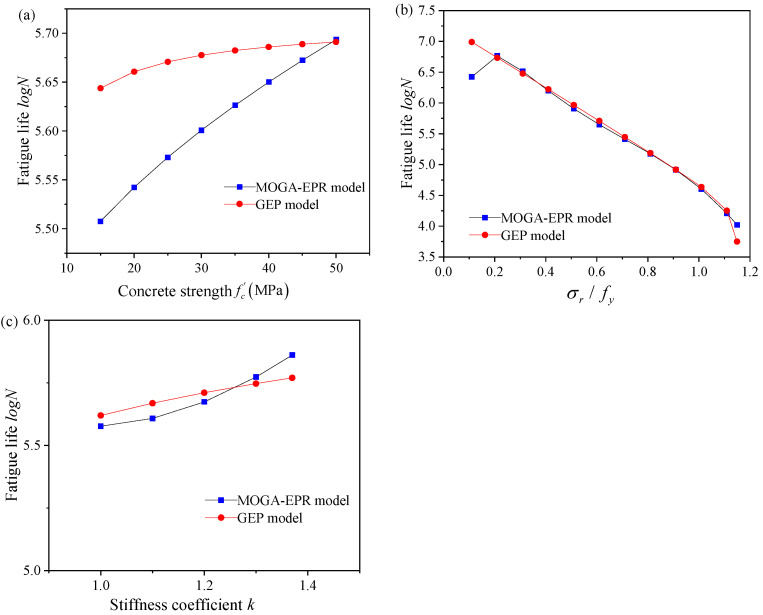
Sensitivity analysis of the model.

A sensitivity analysis was carried out to ascertain whether the GEP and MOGA-EPR models can faithfully capture the correlation between different parameters and fatigue life. When studying the impact of a particular factor on fatigue life, other parameters were held constant and set to their average values, i.e., $f_{c}^{\prime }=34.5,\;\sigma_{r}/f_{y}=0.62,\;k=1.13$. The fatigue life falls with a rise in the ratio of steel reinforcement stress range to yield strength and increases with an increase in concrete compressive strength and stiffness coefficient, as illustrated in Figure 7. This is consistent with the findings in the literature [5,6,9,11,23,24,25,29,30,32,34]. In conclusion, the relationships between different parameters and fatigue life are accurately reflected by the GEP and MOGA-EPR models.

## 4. Comparison of the Proposed Model with Existing Ones

Existing models for predicting fatigue life have been developed mainly by scholars based on their experimental research and by referring to the formulas used for the fatigue life of metal materials. These models primarily reflect the relationship between the steel reinforcement stress range and fatigue life, i.e., the S-N relationship. This paper summarizes some of the fatigue life prediction formulas that are frequently cited and lists them in Table 4. Additionally, Charalambidi et al. [9] proposed a new analysis model for predicting the fatigue life of FRP-strengthened beams using the ratio of the maximum stress to yield strength of longitudinal tensile steel reinforcement and the ratio of axial stiffness of longitudinal tensile steel reinforcement to FRP axial stiffness as variables. To compare it with the model in this paper, it is also included in Table 4.

The GEP and MOGA-EPR models established in this paper are compared with the Papakonstantinou, Dong, Sun, and Charalambidi models in Table 4. The five statistical indicators, including *R*^2^, *RMSE*, *MAE*, *RRMSE*, and *MAPE*, are calculated and presented in Table 5.

As shown in Table 5, both the GEP model and the MOGA-EPR model have a coefficient of determination of 0.8, while other models do not exceed 0.8. Their mean absolute error and other metrics are also lower than those of the other models. The other models only consider the steel stress amplitude, failing to account for the influence of other factors, and the linear regression method may not fully capture the inherent nonlinear characteristics of the problem. In contrast, the GEP model and the MOGA-EPR model comprehensively consider the combined effects of multiple factors and effectively capture the nonlinear relationships in the data, demonstrating higher prediction accuracy and broader applicability.

**Figure 8:**
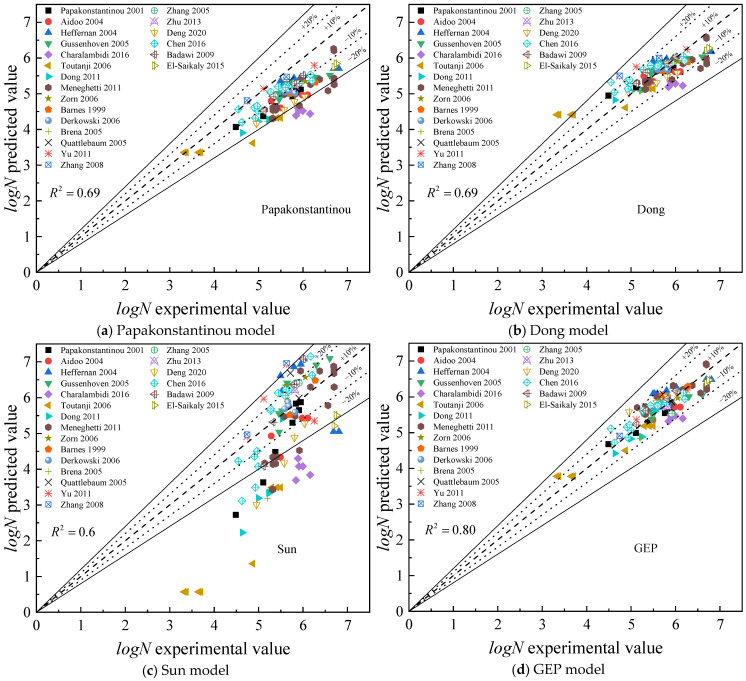

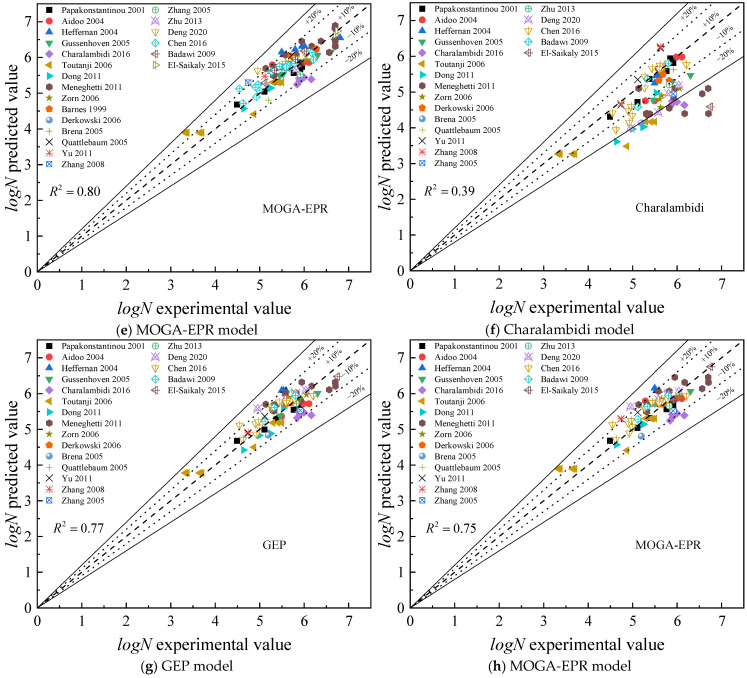
Diagram contrasting the methods used to forecast fatigue life [5,6,10,11,23,24,25,26,27,28,29,30,31,32,33,34,35,36,37,38,39].

Figure 8 compares the predictive results of various models and the experimental outcomes. The Charalambidi model does not provide a calculation formula for cases where $\sigma_{max}/f_{y}<0.6$, and using this model with the database discussed in this article resulted in some pessimistic predictions for the fatigue life of reinforced beams. To facilitate comparison, data under these conditions were excluded, and the analysis was conducted using the remaining 89 data sets, with results displayed in Figure 8f–h. It is visually clear that the Papakonstantinou model, Sun model, Dong model, and Charalambidi model have a large number of data points outside the ±20% error margin, while the data points from the GEP and MOGA-EPR models are concentrated within the ±10% error margin, reflecting the accurate prediction capabilities of the GEP and MOGA-EPR models for the fatigue life of FRP-reinforced beams.

## 5. Conclusions

To solve the current problems of fatigue life prediction of FRP-strengthened reinforced concrete beams, such as model inconsistency, limited computational accuracy, and fewer parameters considered. This paper establishes the fatigue life prediction model by GEP and MOGA-EPR according to extensive experimental data collected, and the following conclusions are drawn:

(1) Utilizing a compiled dataset of 117 samples, five different input forms were proposed based on the input parameters of existing models and the outcomes of Pearson correlation and significance analysis from this study’s database. The programs were evaluated using five indicators to find the optimal input form. The optimal input forms are the ratio of steel reinforcement stress range to yield strength, concrete compressive strength, and stiffness factor.
(2) The GEP and MOGA-EPR models that can predict the fatigue life of FRP-strengthened reinforced concrete beams are developed. The feasibility of the models in this paper is further analyzed by sensitivity analysis. The sensitivity analysis results are consistent with the experimental findings that have been published in the literature, and the models can correctly reflect the effects of each input parameter on the fatigue life.
(3) Comparative analysis of GEP and MOGA-EPR models with existing models revealed that the coefficient of determination for existing models is significantly lower than that of the GEP and MOGA-EPR models, exhibiting lower mean absolute errors among other metrics. The comparison of the predictive values from the GEP and MOGA-EPR models with experimental values shows their high predictive accuracy. This demonstrates that the models developed in this study have significant advantages in capturing nonlinear relationships within the data and are highly applicable for predicting fatigue life.
(4) The GEP model has a more succinct expression form than the MOGA-EPR model.

## Figures and Tables

**Table 1:** Statistical parameters of experimental data.

Parameter	Max	Min	Average	Median	Standard Deviation
*A_s_* (mm^2^)	1962.50	100	429.20	339.30	380
*k_s_* (kN)	392,500	20,100	85,845.70	67,860	76,185
*E_f_* (GPa)	294	20.63	111.37	77.90	71.27
*A_f_* (mm^2^)	390	7.80	99.13	71.20	98.60
*k_f_* (kN)	46,295	408.90	10,348.77	9438.40	9657.80
*f’_c_* (MPa)	50	15.8	34.5	35	7.57
*f_y_* (MPa)	608	335	472	440	74.10
*σ_max_* (MPa)	625.43	176	362	339	95.2
*σ_r_* (MPa)	541.7	67.50	287.1	268	93.5
*σ_r_/f_y_*	1.15	0.12	0.62	0.59	0.22
*σ_max_/f_y_*	1.33	0.44	0.78	0.74	0.21
*k*	1.37	1	1.13	1.11	0.09
*logN*	6.80	3.33	5.63	5.63	0.68

**Table 2:** Software parameter settings.

Parameters	Parameters Setting
Number of genes	3
Number of chromosomes	30
Head length	7
Connection function	+
Mutation probability	0.00138
Permutation probability	0.00546
One-point recombination probability	0.0027
Two-point recombination probability	0.0027
Gene recombination probability	0.0027
Gene transposition probability	0.0027
Constant numbers	10

**Table 3:** Evaluation of fatigue life prediction performance under different input forms.

Model	Input Parameters	*R* ^2^	*RMSE*	*MAE*	*RRSE*	*MAPE* (%)
1	$logN=f_{1}\left(\sigma_{r}\right)$	0.69	0.38	0.32	0.56	5.7
2	$logN=f_{2}\left(f_{y},\sigma_{max}/f_{y},k\right)$	0.72	0.36	0.29	0.53	5.2
3	$logN=f_{3}\left(f_{c}^{\prime },f_{y},\sigma_{r},k\right)$	0.75	0.34	0.28	0.50	5.2
4	$logN=f_{4}\left(f_{c}^{\prime },f_{y},\sigma_{r}\right)$	0.72	0.36	0.28	0.53	5.3
5	$logN=f_{5}\left(f_{c}^{\prime },\sigma_{r}/f_{y},k\right)$	0.80	0.30	0.26	0.45	4.7

**Table 4:** Fatigue life prediction models for FRP-strengthened concrete beams.

Reference	Fatigue Life Prediction Model
Papakonstantinu [10] (2001)	$logN=6.677-0.00613\sigma_{r}$
Dong [11] (2011)	$logN=6.905-0.0046\sigma_{r}$
Sun [12] (2014)	$\left\{\begin{array}{c} logN=10.591-0.0185\sigma_{r}\;\sigma_{r}>175\;\mathrm{MPa}\\ logN=8.217-0.0199\sigma_{r}\;\sigma_{r}\leq 175\;\mathrm{MPa} \end{array}\right.$
Charalambidi [9] (2016)	$\left\{\begin{array}{l} N=\frac{\left[{\left(\frac{\sigma_{max}/f_{y}}{0.00336f_{y}+2.383}\right)}^{\left(1/\left(-0.00019f_{y}-0.04967\right)\right)}\right]}{\left(0.1192\frac{k_{s}}{k_{f}}+0.4798\right)}0.6\leq \sigma_{max}/f_{y}<0.68\\ N=\frac{\left[{\left(\frac{\sigma_{max}/f_{y}}{0.00336f_{y}+2.383}\right)}^{\left(1/\left(-0.00019f_{y}-0.04967\right)\right)}\right]}{\left(-0.0349\frac{k_{s}}{k_{f}}+1.1814\right)}0.68\leq \sigma_{max}/f_{y}\leq 0.78\\ N=\frac{\left[{\left(\frac{\sigma_{max}/f_{y}}{0.00336f_{y}+2.383}\right)}^{\left(1/\left(-0.00019f_{y}-0.04967\right)\right)}\right]}{\left(0.0125\frac{k_{s}}{k_{f}}+1.2983\right)}0.78<\sigma_{max}/f_{y} \end{array}\right.$

**Table 5:** Statistical indicators for various fatigue life prediction models.

Statistical Indicators	*R* ^2^	*RMSE*	*MAE*	*RRSE*	*MAPE* (%)
Papakonstantinou [10]	0.69	0.81	0.72	1.20	12.4
Dong [11]	0.69	0.41	0.31	0.60	5.8
Sun [12]	0.6	1.24	0.95	1.84	18.3
Charalambidi [9]	0.39	0.85	0.65	1.32	11.7
GEP	0.80	0.30	0.26	0.45	4.7
MOGA-EPR	0.80	0.31	0.25	0.46	4.7

## Data Availability

All of the data that is included within the study.
